# Cochlear Implant in Patients with Intralabyrinthine Schwannoma without Tumor Removal

**DOI:** 10.3390/audiolres12010004

**Published:** 2022-01-10

**Authors:** Andrea Laborai, Sara Ghiselli, Domenico Cuda

**Affiliations:** Department of Otorhinolaryngology, Guglielmo da Saliceto Hospital, 29121 Piacenza, Italy; s.ghiselli@ausl.pc.it (S.G.); d.cuda@ausl.pc.it (D.C.)

**Keywords:** intralabyrinthine schwannoma, acoustic schwannoma, cochlear implant

## Abstract

(1) Background: Schwannomas of the vestibulocochlear nerve are benign, slow-growing tumors, arising from the Schwann cells. When they originate from neural elements within the vestibule or cochlea, they are defined as intralabyrinthine schwannomas (ILSs). Cochlear implant (CI) has been reported as a feasible solution for hearing restoration in these patients. (2) Methods: Two patients with single-sided deafness (SSD) due to sudden sensorineural hearing loss and ipsilateral tinnitus were the cases. MRI detected an ILS. CI was positioned using a standard round window approach without tumor removal. (3) Results: The hearing threshold was 35 dB in one case and 30 dB in the other 6 mo after activation. Speech audiometry with bisillables in quiet was 21% and 27% at 65 dB, and the tinnitus was completely resolved or reduced. In the localization test, a 25.9° error azimuth was obtained with CI on, compared to 43.2° without CI. The data log reported a daily use of 11 h and 14 h. In order to not decrease the CI’s performance, we decided not to perform tumor exeresis, but only CI surgery to restore functional binaural hearing. (4) Conclusions: These are the sixth and seventh cases in the literature of CI in patients with ILS without any tumor treatment and the first with SSD. Cochlear implant without tumor removal can be a feasible option for restoring binaural hearing without worsening the CI’s performance.

## 1. Introduction

Schwannomas of the vestibulocochlear nerve are benign, slow-growing tumors, arising from the Schwann cells or neurolemmocytes that support the neurons. Usually, they originate in the internal auditory canal (IAC) or in the cerebellopontine angle cistern (CPA), but they can occur anywhere along the nerve. In the rare case that these tumors originate primarily from the most terminal portion of the eight cranial nerves (neural elements within vestibule, cochlea, or semicircular canals), they can be considered a distinct clinical-pathological disease defined as intralabyrinthine schwannomas (ILSs) [1].

The first case was described in 1917 during an autopsy of a deaf and mentally disabled patient [2], but it was not until the 1970s that there were an intraoperative finding and histopathological description [3]. Until the 1980s, only nine ILSs were described [4]. Since then, thanks to the technological improvements in MRI examination, an increasing number of these tumors have been detected. In the past, ILSs were considered as rare lesions, but currently, they are referred to as an under-reported cause of hearing loss. The use of cochlear implant (CI) for hearing restoration in the case of an inner ear schwannoma is not well defined yet, since there are still many concerns about tumor management, the best surgical approach for tympanic scale access, the possibility of achieving a full electrode insertion, the need for a higher current level for trans-tumoral stimulation, and most of all, the performance obtained and post-operative radiologic surveillance [5]. In our report, we wanted to add to the scant literature on the topic some information about how to manage the hearing restoration in these patients, presenting the first clinical cases in the literature of patients with single-sided deafness that underwent cochlear implant without tumor removal.

## 2. Materials and Methods

### 2.1. Clinical Case 1

A 48-year-old male patient was referred to our department with a 6 year history of left ear complete deafness and ipsilateral tinnitus (score 62 on the Tinnitus Handicap Inventory [6]), due to sudden sensorineural hearing loss. He also complained about non-whirling chronic postural imbalance, especially when standing up. Apart from depression and being in therapy, he did not have any other disease, neurological deficits, or known family history of hearing loss. On pure tone audiometry (125–8000 Hz), there was a profound sensorineural hearing loss of the left ear, while the right hearing was in the normal range. In speech audiometry using 20 phonematically balanced disyllabic words (F. Cutugno, S. Prosser, M. Turrini test) [7], there was no word recognition on the left and a 100% word recognition score on the right side at 30 dB.

In addition to the above-mentioned audiological assessment, a vestibular evaluation was performed. At the bed-side examination using Frenzel goggles, no spontaneous, positioning, or positional nystagmus were detected. The Romberg test was negative, and the smooth pursuit and saccade eye movements were normal. Some abnormalities were detected in the Unterberger test, where a right 80° spin rotation was observed. The Head Shaking Test revealed 2–3 right horizontal nystagmus eye movements. The clinical Head Impulse Test was positive (presence of corrective eye saccades) when rotating the patient’s head towards his left side. The caloric reflex test, performed with hot water (44 °C), showed an areflective left labyrinth. These results were also confirmed in the video Head Impulse Test (vHIT), where a 41% relative asymmetry was detected when the lateral semicircular canals were stimulated, with the left one hyporeflective and with the presence of saccades (gain of 0.61 on the left side and 1.04 on the right). The same pathological result on the left side was obtained at the Suppression Head Impulse Test (SHIMP). The cervical vestibular evoked myogenic potentials were absent on the left side (due to the deafness), while being evocable on the right side until 90 dB.

**Figure 1:**
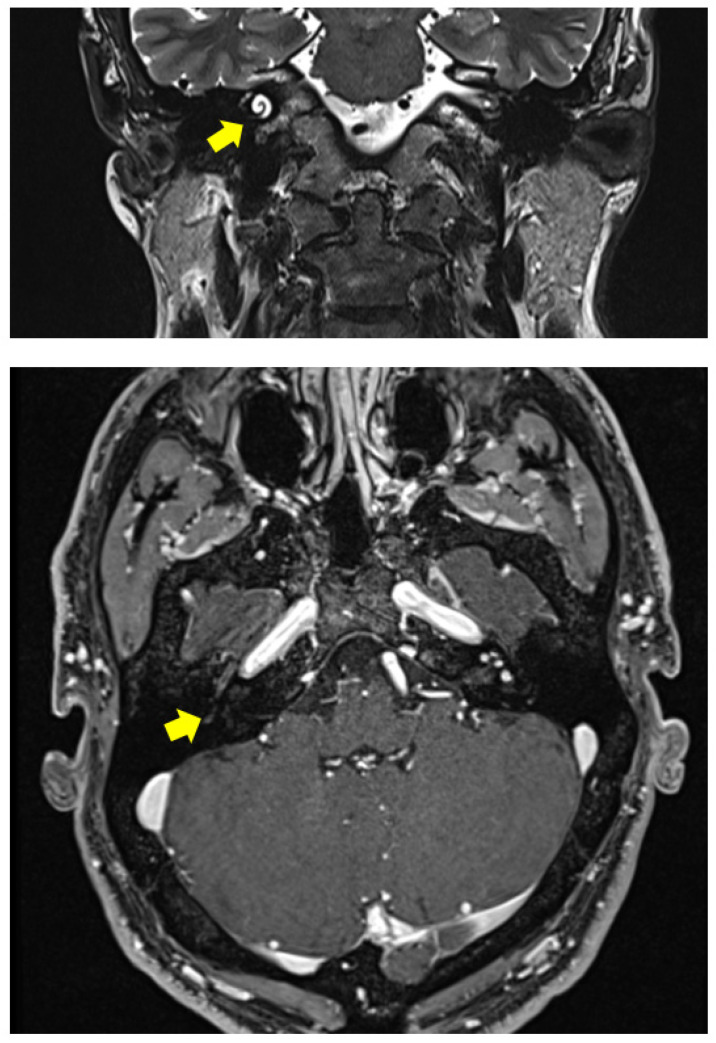
Clinical Case 1: 3 Tesla MRI with gadolinium showing an ILS filling the right cochlea with initial vestibular involvement.

The Dizziness Handicap Inventory score was 42 [8], which corresponds to a moderate grade of disability. A 3 Tesla MRI with gadolinium was carried out, detecting an intracochlear schwannoma completely filling the cochlear turns and with initial extension to the vestibule (Figure 1). The different therapeutic options were explained to the patient and discussed extensively with him (watch and scan, surgical tumor exeresis with concurrent or sequential CI, stereotactic radiosurgery, CI without tumor removal). The patient was determined to restore his hearing, but was afraid of tumor removal and chose the last option.

A CI (Cochlear Model CI512 with a contour advance electrode) was positioned using a standard round window approach. After drilling the round window niche and opening the secondary tympanic membrane, an intracochlear bleeding formation was seen and biopsied through the round window, with histopathological confirmation of an ILS (S100+). The electrode insertion was complete and carried out slowly, with mild resistance aided by a stiff stylet. The intraoperative electrophysiological tests and impedances were normal, and their values remained stable during follow-up. A post-op CT scan was performed, showing a full insertion without tip fold-over.

### 2.2. Clinical Case 2

**Figure 2:**
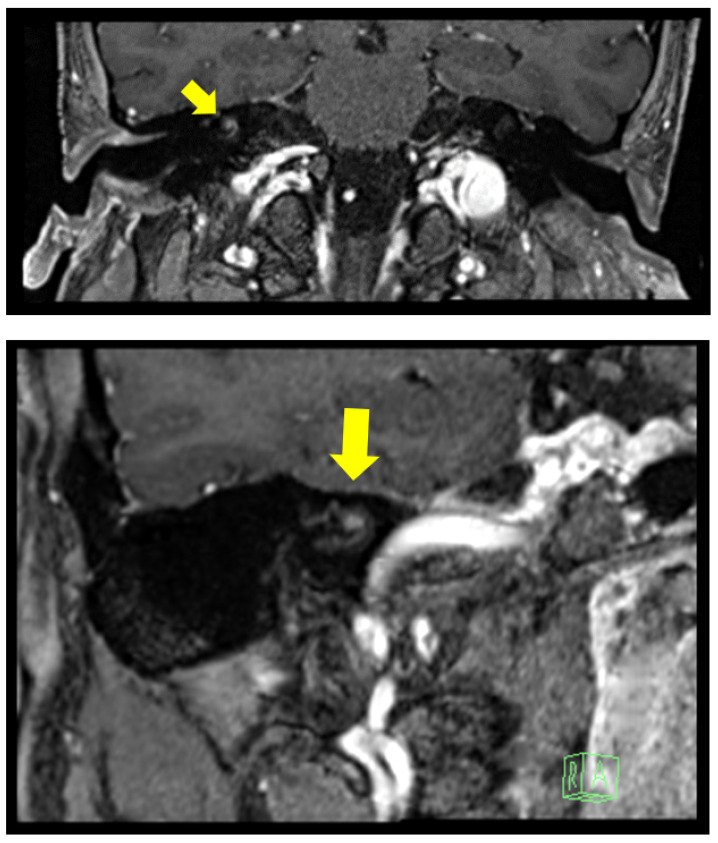
Clinical Case 2: 3 Tesla MRI with gadolinium showing a nodular enhancement, 2.7 mm in diameter, located on the right cochlear basal turn originating from the modiolus.

**Figure 3:**
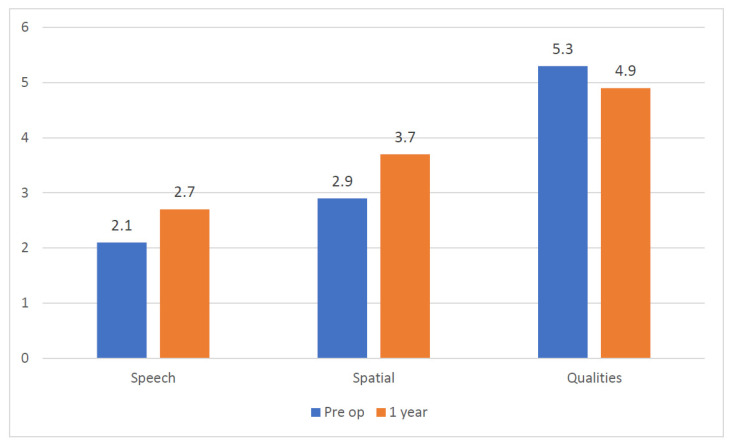
Clinical Case 2: Average SSQ questionnaire score comparison.

The second case was a 50-year-old male patient, with a 1 year history of severe right pantonal hearing loss due to sudden sensorineural hearing loss and normal hearing with just 30 dB sensorineural deficit at 4 kHz and 8 kHz on the left side. He also had sub-continuous right tinnitus and no vertigo. A 3 Tesla MRI with gadolinium showed an ILS located on the cochlear basal turn (Figure 2). He stopped using a hearing aid due to the absence of subjective perceived benefit. The THI score was 36. No word recognition was detected in speech audiometry on the right side (100% word recognition at 50 dB for the left ear). The Italian Matrix Sentence Test (OLSA test) scores are reported in Table 1. The clinical vestibular examination and instrumental tests, as reported for the previous case, were normal. The Speech Spatial and Qualities Questionnaire scores are reported in Figure 3. He was implanted with a Cochlear CI 612. No resistance was reported during electrode insertion, and a biopsy was performed with histopathological confirmation of ILS.

## 3. Results

The first patient complained of some episodes of self-remitting objective vertigo, which occurred during the first post-operative week. At the time of activation, only a slight dizziness was reported. CI was activated 1 mo after surgery using the following parameters (ACE strategy, rate 720 Hz, maxima eight, pulse width thirty), and the implanted ear threshold was 50 dB in the free-field audiometry test. There was no need for a higher current level to stimulate through the tumor compared to standard CI users. We decreased the stimulation rate because it was better tolerated by the patient compared to the usual rate of 900 Hz. Vestibular exams were carried out again with results comparable to the pre-operative ones, both with the implant turned on and off, with just a slight increase in VOR asymmetry in the vHIT (57% vs. 41%) and in the SHIMP (54% vs. 44%). Because of the long-lasting dizziness, we started a vestibular rehabilitation protocol. No facial nerve or other non-auditory stimulation was experienced by the patient.

Tests showed an improvement in the audiological threshold on the implanted ear (40 dB) with a 21% word recognition at 65 dB 1 mo after activation. Dizziness did not change in intensity nor characteristics. The whole test battery was performed again three months and six months after activation. The hearing threshold was 35 dB. Speech audiometry remained the same; however, the tinnitus was completely solved (0 THI score), and the subjective dizziness impairment decreased (12 DHI score), even if the vestibular impairment remained stable at the instrumental examination (asymmetry of 64% in the vHIT and of 65% in the SHIMP). No episode of hearing fluctuation was reported by the patient. A great benefit was observed in the 180° localization test, where a 25.9° azimuth error was obtained when the test was performed with CI on, compared to a value of 43.2° without CI. The data log reported 14 h of daily use.

The second patient reached a 30 dB hearing threshold on the right side 1 mo after activation (ACE strategy, rate 900 Hz, maxima eight, pulse width thirty-seven). He suffered a benign paroxysmal positional vertigo that was solved with one repositioning maneuver. At the last follow-up visit 1 year after the surgery, he reached 27% word recognition at 65 dB on the right ear (contralateral ear masked with an insert). The THI score was 24. The SSQ questionnaire average score improved in the Speech and Spatial items (from 2.1 to 2.7 and from 2.9 to 3.7, respectively), whereas a small decrease was registered in the Qualities area (Figure 3). The average usage time detected by the data log was 11 h per day.

## 4. Discussion

ILSs account for about 10% of all eight cranial nerve schwannomas [9]. The Schwann cells and the myelin sheaths of cochlear axons are distally extended until the modiolus, proximal to the spiral ganglion. ILSs usually arise in the modiolus and then may have a three-way spreading pattern: cochlear basal turn invasion, cribriform area erosion, or intravestibular diffusion [10]. To the best of our knowledge, less than one thousand cases have been described [11]. ILSs are an underestimated cause of vertigo and hearing loss, but thanks to the technological improvement in the imaging technique, especially MRI, an increasing number of these tumors may be detected, also at a very small size (2–3 mm), and are becoming important in the differential diagnosis of cochleovestibular disorders [12].

The first ILS classification was proposed by Kennedy et al., in 2004 [1], with a seven group division according to their radiological extent within the labyrinth: intravestibular, intracochlear, vestibulocochlear, transmodiolar, transmacular, transotic, tympanolabyrinthine. A subsequent modification to this scheme was made in 2013 by Van Abel et al. [13] and is still the most recent one. They renamed these lesions as primary inner ear schwannoma and added another category to include the ILSs with extension to the CPA (named “+CPA”). The intracochlear location seems to be the most common [11].

ILS’s symptoms are aspecific and mimic the ones typical of Mèniére’s syndrome. Hearing loss is present in almost all patients (>95%) with different types of onset, from sudden (15–32%) to slowly progressive and also fluctuating [11]. Hearing loss is not always sensorineural. A mixed type can occasionally be present [12], due to the conductive component secondary to stapes movement interference caused by tumor pressure on the footplate inner surface [1]. Another possible explanation is secondary endolymphatic hydrops that may cause also dizziness and ear fullness (2%). The same hearing symptom heterogeneity can be found in the balance problems, where dizziness (35%), spinning vertigo (22%), and postural instability have been reported. Furthermore, vestibular symptoms are more likely to be found in patients with ILS than in cases of schwannomas located elsewhere along the nerve [13].

Despite an increasing awareness of ILSs, the delay in diagnosis seems to be still significant with a mean diagnostic delay varying from 3–8 year, according to the clinical symptoms, where hearing loss has significantly shorter delays than vertigo [14].

It is still unclear whether there is a correlation between the involved site and the symptoms. Salzman et al. [15] reported in their series that tumor location had no reproducible bearing on the symptoms, but recently, Elias et al. [11] described a case in which a strong correlation seemed to exist.

MRI with gadolinium is the gold standard exam for diagnosis. The first MRI description of ILSs was published by Mafee et al. in 1990 [16]. ILSs appear as a hypointense filling defect inside the labyrinth with replacement of the normal high-signal-intensity fluid (signal void) on high-resolution T2-weighted images or as focally enhanced masses with sharp borders on T1 sequences. The radiological differential diagnosis has to be posed with labyrinthitis, hemorrhage, or ossification [15].

As for all schwannomas, also ILSs seem to have a very slow growth tendency, with a different growing rate reported in the literature, varying from 15–59% [9,10,11,12,13,14,15,16]. Furthermore, they are surrounded by the otic capsule and therefore have a strong obstacle for their growth. The conservative “watch and scan” therapeutic strategy seems to be the most common approach (56%) [11], especially for small intracochlear ILSs with a serviceable hearing. We suggested this option to our patients, but their main concern was to restore their hearing. Because of this, in order to preserve the cochlear anatomy, due to the absence of intractable vertigo, and mainly for the patients’ fear and strong refusal, we decided not to perform tumor exeresis, but only CI surgery to restore a functional binaural hearing, without the possibility of decreasing the CI performances due to fibrosis, scaring, and limited extension for array insertion, which would have been a possible consequence of a subtotal cochleotomy.

The first case of tumor removal and cochlear implantation was described by Kronenber et al. in 1999 [17].

Many papers about tumor exeresis and hearing rehabilitation by CI in ILS patients have since been published, describing both synchronous or staged surgeries, where a dummy electrode is placed inside the cochlea after tumor removal and before CI insertion, in order to prevent cochlear ossification and subsequent obliteration. In our opinion, ILS tumor exeresis should be performed only in the case of the initial extension into the IAC or when vestibular untreatable symptoms are present.

In the case series described by Plontke [18], after tumor removal and CI, an average 33% word recognition score was obtained in the monosyllabic test after 6 mo of follow-up. This result is comparable to the one obtained for our patients, also taking into account that we used a more difficult disyllabic word test.

Aschendorff et al. reported a group of eight implanted patients in which the tumor was removed. Considering the four with at least 6 mo of audiological follow-up, the average score at 65 dB for monosyllabic word recognition was 39%, with a wide range varying from 10% to 95% [19].

To the best of our knowledge, there is only one other report of CI without ILS removal, where ten patients were described, but only five had never received any treatment before implantation [5]. Despite that tumor presence can be an obstacle to electrode insertion, in our cases, no resistance was reported, while Carlson noticed it in 5/10 patients, with one requesting a device substitution after an unsuccessful insertion attempt, and a tip fold-over occurred in another case. No higher current levels were required for trans-tumoral stimulation. No facial nerve stimulation was reported. As for our cases, CI seems to be a feasible and effective option in patients with ILSs, when the cochlear nerve is intact. Carlson reported an average 48.8% of open-set word recognition with 9.8 mo mean follow-up in his series of untreated patients. In our case, we obtain a 21% word recognition score at 6 mo of follow-up in one case and 27% after 1 year in the other one. We believe that our inferior result can be explained mainly by two points: firstly, our patient had single-sided deafness with normal hearing on the contralateral ear, and secondly, there was a shorter audiological follow-up time. We are confident that the word recognition score will hopefully continue to improve as the first patient continues his auditory training. In fact, if we take into account in the Carlson series only the patients with a follow-up shorter than 1 year, we obtain a mean word recognition score of 36.6%.

Stereotactic radiosurgery seems to have an indication only in very selected cases: older or inoperable patients with a growing tumor. Plontke et al. [18] suggested this because of the good surgical accessibility of ILSs and the possible damage from radiation to the sensorineural structures, which may lead to unfavorable functional prognosis, especially concerning CI rehabilitation. Other possible adverse side effects of this therapy are facial nerve damage, malignant transformation, and unclear tumor control. Carlson et al. [5] reported four patients treated with radiotherapy and subsequently implanted: with a mean follow-up of 8.5 mo, their average word recognition score was 45%.

A recent study described eight patients affected by neurofibromatosis 2 and ILS that were treated by CI without tumor removal. They described a wide range of results (from 0–100% for the sentence test), with higher current levels of stimulation required for patients previously treated with radiotherapy [20]. According to the literature, for correct follow-up, an MRI should be performed 1 year post-diagnosis (1.5 T) and repeated every 2 year if no growth can be seen [5]. This protocol is applicable also to studying the inner ear of patients with CI, by placing the electrode in an exaggerated posterosuperior location in order to avoid artefacts on the inner ear and the CPA and using a 2D sequence instead of a 3DMRI sequence (Drive, CISS), for better viewing the labyrinth and the IAC. By doing this, these anatomical structures can be correctly seen in at least one sequence [21]. The technological development of CI has led to the creation of devices that are 3T MRI compatible. This could be an important step forward in the management of these patients, since we can perform a more accurate follow-up, especially for these very small lesions.

The limitations to our study were the small number of patients, the possibility of a difficult schwannoma follow-up (but this can be solved using the abovementioned technique), the limited follow-up time, and the absence of a comparison with audiological results of patients treated by radiosurgery and then IC, but to the best of our knowledge, no study exists about this therapeutic approach.

## 5. Conclusions

These two cases are the sixth and seventh cases in the literature of CI in patients with ILS without any tumor treatment, but the first concerning single-sided deafness. MRI is mandatory in the case of asymmetric progressive or sudden hearing loss. Apart from the CPA and the intracanalicular portion of the eight cranial nerves, attention must be paid also to the labyrinth in order to rule out the presence of an ILS. Cochlear implant without tumor removal can be a feasible option for restoring binaural hearing without worsening the CI’s performances, also in the case of single-sided deafness.

## Figures and Tables

**Table 1:** Clinical Case 2: Italian Matrix Sentence Test SNR scores at the pre-operatory evaluation and one year after cochlear implant. S0 refers to sound coming from a source in front of the patient, Nic to noise coming from 90° on the implanted side, and Nnh to noise coming from 90° on the normal hearing side.

	Pre-Op Evaluation	1 Year
**S0 N0**	−3.6	−4.8
**S0 Nic**	−9	−8.5
**S0 Nnh**	−0.1	−1.9

## Data Availability

Raw and extended data are available upon request from the corresponding author.
